# Clinical Implications of Exosomal PD-L1 in Cancer Immunotherapy

**DOI:** 10.1155/2021/8839978

**Published:** 2021-02-08

**Authors:** Sergio Ayala-Mar, Javier Donoso-Quezada, José González-Valdez

**Affiliations:** Tecnologico de Monterrey, School of Engineering and Science, Av. Eugenio Garza Sada 2501 Sur, Monterrey, NL, Mexico

## Abstract

Inhibiting the programmed cell death ligand-1 (PD-L1)/programmed cell death receptor-1 (PD-1) signaling axis reinvigorates the antitumor immune response with remarkable clinical efficacy. Yet, low response rates limit the benefits of immunotherapy to a minority of patients. Recent studies have explored the importance of PD-L1 as a transmembrane protein in exosomes and have revealed exosomal PD-L1 as a mechanism of tumor immune escape and immunotherapy resistance. Exosomal PD-L1 suppresses T cell effector function, induces systemic immunosuppression, and transfers functional PD-L1 across the tumor microenvironment (TME). Because of its significant contribution to immune escape, exosomal PD-L1 has been proposed as a biomarker to predict immunotherapy response and to assess therapeutic efficacy. In this review, we summarize the immunological mechanisms of exosomal PD-L1, focusing on the factors that lead to exosome biogenesis and release. Next, we review the effect of exosomal PD-L1 on T cell function and its role across the TME. In addition, we discuss the latest findings on the use of exosomal PD-L1 as a biomarker for cancer immunotherapy. Throughout this review, we propose exosomal PD-L1 as a critical mediator of tumor progression and highlight the clinical implications that follow for immuno-oncology, discussing the potential to target exosomes to advance cancer treatment.

## 1. Introduction

The success of cancer immunotherapy highlights how the maintenance and expansion of malignancy strongly depend on immunosuppression [1]. The PD-L1/PD-1 signaling pathway is a highly conserved immune checkpoint that under physiological conditions mediates immunotolerance [2]. However, tumor cells harness immune checkpoints for immune evasion, which ultimately leads to tumor survival, growth, and invasion [1].

Over the last decade, the US Food and Drug Administration (FDA) has approved six human monoclonal antibodies that prevent PD-L1/PD-1 binding and reinvigorate an exhausted antitumor inflammatory response with remarkable clinical efficacy [3, 4]. Nonetheless, low response rates and therapy resistance limit the benefits of immune checkpoint blockade to a minority of patients [5].

Recent evidence suggests that adaptive cancer responses mediate changes in PD-L1 expression and subcellular localization, which have been associated with therapy failure [6]. Indeed, tumor cells establish a complex network of intercellular communication that exhibits a constant evolution [7]. Tumor cells modulate signaling pathways throughout the TME either by direct cellular contact or the secretion of soluble factors, such as signaling molecules and extracellular vesicles (EVs) [8]. Among EVs, exosomes have been associated with the establishment and maintenance of the TME through cancer-associated differentiation and immunomodulation [9, 10]. Exosomes have a size range of 30 to 150 nm and are composed of a lipidic bilayer that encloses a specific cargo of proteins and genetic material [11].

Most recently, exosome signaling has been directly associated with immune checkpoint blockade therapy [8]. Evidence suggests that PD-L1, which is found at the cellular membrane, is also secreted as an exosomal transmembrane protein [12]. PD-L1 mRNA and DNA, as well as small RNA species that regulate PD-L1 expression, have also been found enclosed in tumor-derived exosomes [13–15]. Remarkably, homotypic and heterotypic exosome transfer also regulates PD-L1 expression across the TME [14, 16].

It is now known that exosomal PD-L1 elicits the same function as its cellular counterpart and binds with the same affinity to PD-1 and even to monoclonal antibodies against PD-L1 [14, 17]. In light of these findings, exosomal PD-L1 has been suggested as a primary mediator of immune escape and tumor progression, as well as a mechanism of therapy resistance. Even more, the level of circulating exosomal PD-L1 has been proposed as a biomarker to predict and evaluate immunotherapy response [18, 19].

Here, we review the role of exosomal PD-L1 in cancer immunotherapy; accordingly, we discuss the immunological mechanisms by which exosomal PD-L1 is proposed to mediate immune escape and the clinical implications that follow for cancer treatment. Furthermore, we review recent data on the use of exosomal PD-L1 as a predictive biomarker for immunotherapy and discuss potential therapeutic approaches that intend to target exosomes in the TME.

## 2. Immunological Mechanisms of Exosomal PD-L1

### 2.1. Biogenesis and Release

Exosomes are complex shuttles for intercellular communication which is critical for the establishment and maintenance of a protumoral microenvironment [20]. Exosomal PD-L1 appears to be at the crossroads of inflammation and tumor progression, and thus, elucidating the factors and mechanisms that lead to its biogenesis and release is needed to fully understand how tumor cells harness the inflammatory response during malignant evolution.

Available data shows that exosomal PD-L1 originates from the plasma membrane, rather than from the endoplasmic reticulum or Golgi apparatus [17, 21, 22]. Accordingly, early endosomes that form by endocytosis of the cellular membrane might be the source of exosomal PD-L1. Evidence suggests that PD-L1 is distributed amongst different cellular compartments, which have been associated with immunotherapy failure [23]. However, the regulatory mechanisms that dictate PD-L1 distribution and exosomal PD-L1 biogenesis are not completely understood.

In this setting, PD-L1 has been found to be colocalized with tetraspanin CD63, a classical exosomal marker involved in intracellular vesicular transport, and with proteins of the endosomal sorting complexes (ESCRT) in tumor tissue [16, 17]. For instance, PD-L1 colocalizes with the hepatocyte growth factor regulated tyrosine kinase substrate (HRS), a member of ESCRT-0 required for early exosomal cargo recognition and sorting [17]. HRS recognizes monoubiquitylated cargo proteins and begins the sequential recruitment of ESCRT subunits; the deletion of this ESCRT member in melanoma cells decreases exosomal PD-L1 and increases cell surface PD-L1 [12, 24].

Interestingly, Mezzadra et al. found that CMTM6 (CKLF- (chemokine-like factor-) like MARVEL transmembrane domain-containing family member 6) interacts molecularly with PD-L1 at the cell surface, decreasing its ubiquitination and increasing PD-L1 stability [22]. CMTM6 knockout results in a reduction of PD-L1 surface levels, without affecting transcription levels [22]. A hypothesis derived from the aforementioned findings is that exosomal PD-L1 levels may be increased after CMTM6 knockout. Accordingly, addressing the association between PD-L1 posttranslational regulation and members of the ESCRT complex could shed light on the mechanisms that regulate PD-L1 distribution at the subcellular level.

The ESCRT-accessory protein ALIX has also been associated with PD-L1 expression at the cell and exosomal surface [25]. Monypenny et al. found that ALIX depletion in breast cancer cells reduces exosomal PD-L1 release and increases cell surface PD-L1 expression while upregulating oncogenic signaling through epidermal growth factor receptor (EGFR) activity [25]. These findings suggest that the exosomal PD-L1 release occurs parallel to cell surface PD-L1 expression and that ESCRT-accessory proteins control the PD-L1 distribution between the cell and exosome membranes [25]. Therefore, since there appears to be an association between PD-L1 distribution, exosome biogenesis, and tumor growth, the syndecan-syntenin-ALIX axis can be proposed to have major implications in immunotherapy.

On its part, exosome secretion is strongly dependent on neutral sphingomyelinase 2 (nSMase2) and Rab27a, which are involved in intravesicular vesicle budding and fusion of the multivesicular bodies to the plasma membrane, respectively [26, 27]. The experimental knockout of both nSMase2 and Rab27a in cancer cell lines inhibited exosomal PD-L1 release [17]. Moreover, inhibiting nSMase2 activity also induced a decline in PD-L1 transcription [17]. Conversely, the experimental deletion of PD-L1 in PC3 cells did not alter exosome secretion [17]. In this sense, the available evidence indicates that PD-L1 may be sorted into exosomes from the plasma membrane, yet additional questions that remain to be addressed include how other mechanisms of endosome maturation and exosome release influence exosomal PD-L1 biogenesis.

As previously noted, the experimental deletion of members of the ESCRT complex and accessory proteins inhibits exosomal PD-L1 release and increases PD-L1 expression at the cell surface [17, 25]. Kim et al. found that in lung cancer cell lines the amount of exosomal PD-L1 in the culture supernatant represented the amount of PD-L1 expression on the cell surface, while the abundance of exosomal PD-L1 isolated from plasma of non-small-cell lung cancer (NSCLC) patients correlated with tumor PD-L1 positivity [28]. Certainly, differential PD-L1 expression among tumor types is one of the major hurdles of immunotherapy, and there seems to be an association between the amount of PD-L1 expressed at the cell surface and the exosomal membrane [1, 25]. The nature of the aforementioned association and the factors that may induce a PD-L1 shift from the cell surface to the exosome membrane require further study.  Table 1 presents changes in PD-L1 subcellular expression upon cytokine stimulation or inhibition of regulatory proteins of exosome biogenesis and release in a variety of cancer cell types.

Even more, the extent of exosomal PD-L1 secretion may explain differences in the heterogeneous cell surface PD-L1 expression pattern observed among different types of tumors [17]. Chen et al. used a panel of human melanoma cell lines to demonstrate that the level of exosomal PD-L1 is higher in metastatic than in primary tumors, suggesting that the dynamic PD-L1 distribution at the subcellular level and the release of exosomal PD-L1 may be associated with the metastatic capacity of tumor cells [12].

Finally, other forms of extracellular PD-L1 are also found in the systemic circulation; for instance, circulating PD-L1 in microvesicles or its membrane-free soluble forms have been identified [18, 30]. Research into the biological significance of other forms of soluble PD-L1 and the mechanisms that lead to their biogenesis and secretion may also improve immunotherapy efficacy.

### 2.2. Exosomal PD-L1 Regulatory Factors

PD-L1 is expressed in tumor cells as a result of constitutive oncogenic signaling, genomic aberrations, epigenetic alterations, and microenvironmental factors, such as proinflammatory signaling and hypoxia [31]. As an example, interferon (IFN) receptor signaling upregulates tumor PD-L1 expression; however, it is not known if regulatory factors that induce PD-L1 expression increase as well exosomal PD-L1 release levels [32].

Interestingly, Chen et al. found that in patients with metastatic melanoma, the level of circulating exosomal PD-L1 correlates to that of serum IFN-*γ* and that exosomes derived from melanoma cells treated with IFN-*γ* exhibited enhanced binding to PD-1 *in vitro* [12]. In this setting, exosomal PD-L1 release could be thought of as a reciprocal immunosuppressive mechanism in response to IFN-*γ* secreted by CD8 T cells, macrophages, and natural killer (NK) cells [33].

Further evidence supports the assumption that PD-L1 release is partly mediated by cytokine induction, as shown by an increase in exosomal PD-L1 release in response to IFN-*α*, IFN-*γ*, and TNF-*α* in melanoma and glioblastoma cells [15, 29]. Additional cytokines, such as TGF-*β*1 and IL-17, have also been associated with PD-L1 expression; however, their role in exosomal PD-L1 release is not fully understood [31, 34].

For instance, Porcelli et al. demonstrated that mast cells induce the release of TGF-*β*1 and chemotherapy resistance in pancreatic cancer cells *in vitro*, through a mechanism dependent on ERK1/2 and Akt signaling activation, which are common in pancreatic cancer because of KRAS mutations [35]. Even more, ERK1/2 and AKT are also downstream mediators of PD-L1 expression, further suggesting that the immune response and tumor mutational alterations play a pivotal role in the development of a complex network of cytokine signaling that results in the expression of immunomodulatory molecules in cancer cells [36].

**Figure 1:**
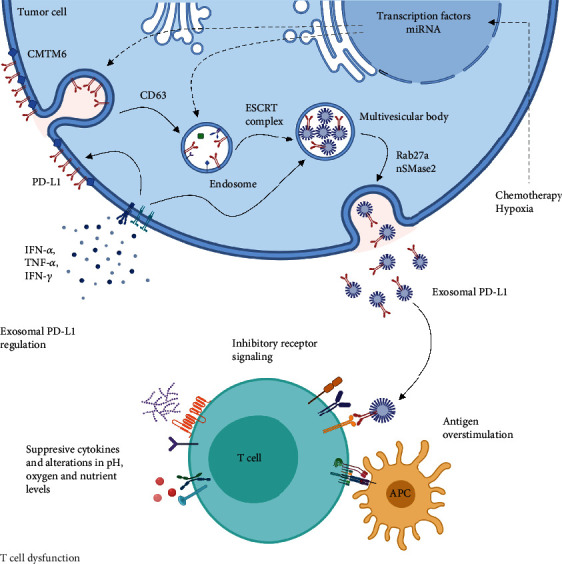
Model of potential biogenesis and release of exosomal PD-L1 and its role in T cell dysfunction. Cytokine signaling in tumor cells increases PD-L1 expression and exosomal PD-L1 biogenesis which is mediated by intracellular vesicular transport proteins, such as CD63 and the endosomal sorting complex required for transport (ESCRT) machinery. Other factors such as hypoxia and chemotherapy may induce exosomal PD-L1 biogenesis, by enhancing the activity of transcription factors and small RNA species via unknown mechanisms (dotted arrows). Rab27a and nSMase2 mediate exosomal PD-L1 release which contributes to T cell dysfunction by inhibitory receptor signaling, in the context of multiple immunosuppressive factors in the TME.

Cytokine induction of exosomal PD-L1 release may thus reflect the interplay between the tumor and the immune system (Figure 1). Nonetheless, tumor immune escape partly results from adaptive tumor responses to a variety of factors. [31]. Microenvironmental factors associated with immunosuppression include alterations in pH, nutrients, and oxygen concentration [37]. In this regard, tumor hypoxia has been recognized as a powerful driving force of immune escape and tumor progression [38].

The response to hypoxia is mainly mediated by hypoxia-inducible factors (HIF) since low oxygen concentrations induce the stabilization and nuclear translocation of HIF-1*α* which heterodimerizes with HIF-1*β* to form the HIF1*α*/*β* complex [38]. The HIF1*α*/*β* complex cooperates with signal transducer and activator of transcription 3 (STAT3) to upregulate PD-L1 expression [38, 39].

Hypoxia in the TME also increases exosome release in a HIF-1*α*-dependent manner [40]. However, the release of exosomal PD-L1 as a mechanism of tumor hypoxic evolution has not been thoroughly explored. Hypoxia-induced exosome release has been associated with Rab27a function which has also been associated with exosomal PD-L1 biogenesis [23, 27, 40]. Thus, hypoxia may be a promoter of exosomal PD-L1 release and sets yet another experimental setting in which PD-L1 subcellular distribution can be studied. Furthermore, downregulating the response to hypoxia by inhibiting HIF-1*α* and STAT3 can result in both PD-L1 and exosome inhibition.

Along with microenvironmental factors, therapeutic interventions can also promote exosomal PD-L1 biogenesis and release. Evidence indicates that the chemotherapeutic agent 5-fluorouracil (5-FU) increases tumor-derived exosomal PD-L1 release via the miR-940/Cbl-b/STAT5a axis in patients with extensive-stage gastric cancer [41]. Likewise, Dosset et al. found that immunocyte-derived cytokines may increase PD-L1 expression after chemotherapy in colon cancer [42].

Auspicious results have been achieved by developing combinatorial strategies employing conventional chemotherapeutics and immune checkpoint blockade. However, a variety of cancer types do not respond to these strategies [43, 44]. In this context, cytokine signaling and small RNA species may mediate PD-L1 expression and exosomal PD-L1 release hindering the clinical efficacy of novel combinatorial approaches. Yet, further research is required to fully elucidate the regulatory mechanisms that result in PD-L1 and exosomal PD-L1 upregulation after conventional therapeutics.

Recent evidence shows that exosomal PD-L1 may mediate primary resistance to antibodies against PD-L1 and that the level of circulating exosomal PD-L1 changes during the course of anti-PD-1 therapy [17]. Furthermore, inhibiting exosomal PD-L1 release improves immunotherapy response, even in the context of primary resistance [17]. These findings strongly suggest that exosomal PD-L1 release is an adaptive cancer response and may thus be a critical mediator of malignant evolution.

In this context, exosomal PD-L1 appears to be a potential therapeutic target that may improve both conventional and novel therapeutic approaches. For this purpose, research is starting to focus on downregulating the PD-L1 expression or exosome release, rather than blocking PD-L1/PD-1 interaction [2]. Identifying the factors that upregulate exosomal PD-L1 is crucial for the development of novel therapeutic molecules. Indeed, targeted immunotherapy employing the combination of immune checkpoint blockade and small molecular inhibitors has shown promising results, as demonstrated by Mariathasan et al., who found that concomitant TGF-*β* inhibition and immune checkpoint blockade enhances immunotherapy efficacy in a mouse mammary carcinoma model [45, 46].

Exosome inhibitors include GW4869, imipramine, D-pantethine, Y27632, manumycin A, and calpeptin and have been recently reviewed elsewhere [47]. It should be noted that blocking exosome secretion could attenuate extracellular PD-L1 release. However, developing strategies to suppress the activity of the ESCRT complex or accessory proteins must be done with caution because of the relationship between the PD-L1 expression at the cell surface and the exosome membrane (Table 1). Certainly, exosome inhibitors provide an opportunity to further assess PD-L1 subcellular distribution.

### 2.3. The Role of Exosomal PD-L1 on T Cell Dysfunction

In the setting of tumor immune escape, T cells have acquired an exhausted phenotype due to continuous antigen-mediated activation, inhibitory receptor signaling, metabolic dysfunction, and other microenvironmental and tissue factors [48]. The use of immune checkpoint inhibitors (ICIs) is a new standard of care for advanced tumors, mainly because inhibiting the PD-L1/PD-1 axis reinvigorates T cell effector function which includes direct cytotoxic activity against tumor cells [49, 50].

Cell-cell interactions mediate T cell exhaustion by inhibitory receptor signaling [50]. In this context, the expression of PD-1 is dependent on the context of the inflammatory microenvironment and can be observed across a variety of hematopoietic cells [1]. T cells show low basal levels of PD-1 expression, which transiently increase upon antigen-associated activation to then return to basal levels after antigen clearance [51]. However, chronic antigen-associated stimulation results in a sustained increase of the PD-1 expression, a hallmark of T cell exhaustion [52]. Even more, PD-1 appears to be required for regulatory T cell and follicular helper T cell development [51].

Upon binding to PD-L1, PD-1 undergoes a conformational change that induces the phosphorylation of the immunoreceptor tyrosine-based inhibitory motif (ITIM) and the immunoreceptor tyrosine-based switch motif (ITSM), leading to the recruitment of cytoplasmatic SHP-1 and SHP-2 protein tyrosine phosphatases [1]. Subsequently, SHP-1/2 prevents the phosphorylation of intracellular mediators of the PI3K/AKT/mTOR and MAPK signaling pathways and thus terminate T cell activation [1, 53].

**Figure 2:**
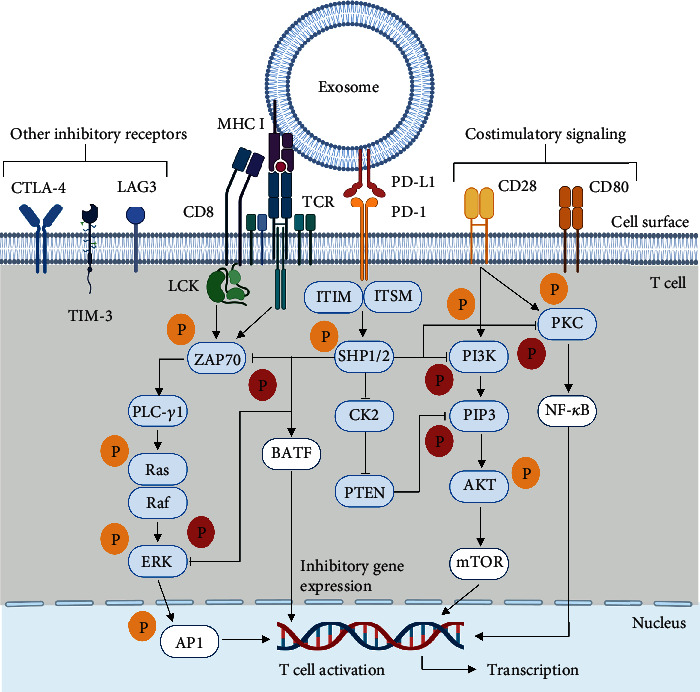
The proposed inhibitory signaling in T cells mediated by exosomal PD-L1. Upon antigen presentation, the T cell receptor (TCR) and costimulatory receptors CD8, CD28, and CD80 mediate the phosphorylation of intracellular mediators (blue boxes) of the PI3K/AKT/mTOR and MAPK signaling pathways which results in the activation of transcription factors (white boxes) that induce T cell activation. Upon binding of exosomal PD-L1 to PD-1, SHP-1 and SHP-2 protein tyrosine phosphatases prevent the phosphorylation of PKC, PI3K, ZAP70, and ERK, thereby inhibiting T cell effector function. SHP-1/SHP-2 also inhibits CK2, allowing phosphatase and tensin homolog (PTEN) to prevent PIP3 phosphorylation. SHP-1/SHP-2 are also proposed to mediate inhibitory gene expression via basic leucine zipper ATF-like transcription factor (BATF). Other inhibitory receptors also mediate T cell dysfunction in the context of tumor immune escape.

Available data shows that PD-L1 as a transmembrane protein in exosomes elicits the same function as its cellular counterpart (Figure 2) [19]. In this line, Chen et al. found that exosomal PD-L1 exhibits higher interaction with activated T cells [12]. Consequently, exosomal PD-L1 may contribute to an immunosuppressive TME by terminating T cell activation and sustaining T cell exhaustion [14, 54]. As previously noted, T cells express PD-1 as a result of both, acute and continuous antigen-associated activation, a process mediated by the T cell receptor (TCR), along with costimulatory signaling [55].

To demonstrate that exosomal PD-L1 inhibits TCR-mediated T cell activation, Ricklefs et al. tested the effect of glioblastoma-derived exosomes in the activation and proliferation of peripheral blood mononuclear cells (PMBCs) stimulated with anti-CD3 and dendritic cell-mediated antigen presentation [15]. In this experimental model, exosomal PD-L1 inhibited the CD4+ and CD8+ T cell proliferation and decreased the expression of CD69 and CD25, early and late activation markers, respectively [15].

PD-1 inhibits TCR signaling to terminate T cell activation, which requires the interaction of the TCR and costimulatory receptors with major histocompatibility complex (MHC) molecules [56, 57]. Tumor-derived exosomes also exhibit sustained expression of MHC molecules in their surface and thus provide the proper platform for PD-1 function [24, 58]. It should be noted that T cell activation also requires costimulatory receptor signaling [48].

To test if exosomal PD-L1 inhibits CD3/CD28-mediated T cell activation, Yang et al. assessed the phosphorylation and activity of intracellular mediators of costimulatory signaling [16]. Accordingly, evidence shows that breast cancer-derived exosomal PD-L1 downregulates ERK phosphorylation and NF-*κ*B activation in PBMCs treated with phytohemagglutinin (PHA) [16]. Thus, in addition to inhibiting TCR signaling, exosomal PD-L1 may also terminate T cell activation by blocking CD3/CD28 downstream signaling.

Recent evidence suggests that there might be an association between the levels of circulating exosomal PD-L1 and CD28 expression in CD8+ T cells in various advanced tumors [59]. Current studies indicate that exosomal PD-L1 is able to attenuate both, TCR and costimulatory receptors signaling, which is not trivial since these receptors exhibit distinct expression patterns [48].

In this context, immune checkpoint blockade reinvigorates T cell effector function by enabling T cell activation which is required for cytokine production [57]. T cell effector function is characterized by the production of interferon-*γ* (IFN-*γ*), tumor necrosis factor (TNF), and IL-2, as well as high proliferative capacity and cytolytic degranulation [48]. During T cell exhaustion, effector functions are lost in a hierarchical manner; first, IL-2 production and proliferation cease, followed by deficient degranulation, and IFN-*γ* and TNF release [60].

Evidence shows that in the setting of Raji B cell antigen presentation to Jurkat T cells, exosomal PD-L1 from PC3 cells decreases IL-2 secretion [17]. Furthermore, exosomal PD-L1 has been shown to also decrease IFN-*γ*, TNF-*α*, granzyme B, and perforin T cell secretion *in vitro* [16, 28, 61]. Consequently, tumor cells may counteract CD8+ T cell function at its effector stage, preventing cytokine production and cytolytic degranulation without the need of cell-cell interactions.

In addition to suppressing T cell cytokine release, exosomal PD-L1 has shown to decrease T cell proliferation and to increase T cell apoptosis both *in vivo* and *in vitro* [12, 17, 28, 62]. It is worth noting that the degree of tumor infiltration by T cells is critical for antitumor immunity and has been proposed as a predictor of immunotherapy response [63]. Evidence shows that by inhibiting proliferation and inducing apoptosis, exosomal PD-L1 decreases the number of tumor-infiltrating T cells *in vivo* [12, 28].

Remarkably, PD-L1 as a transmembrane protein in exosomes has the potential to regulate T cell function and differentiation beyond the TME. Poggio et al. found that exosomal PD-L1 decreases the number of CD4+ and CD8+ T cells while sustaining FoxP3 regulatory T cells in the lymph node [17]. Even more, cancer cells that are unable to secrete exosomal PD-L1 induce long-term systemic immunity [17]. Thus, exosomal PD-L1 may be a critical mediator of systemic immunosuppression, promoting an environment suitable for metastasis [17].

The aforementioned findings suggest that PD-L1 in exosomes effectively terminates T cell activation, inhibits T cell effector function, and decreases the number of T cells, inducing immunosuppression in the TME and systemically. Nonetheless, the clinical efficacy of immune checkpoint blockade demonstrates that T cell exhaustion is a reversible process rather than a definitive phenotype [48]. Available data shows that the effect of exosomal PD-L1 on T cell function can be prevented or reversed employing antibodies against PD-L1 or PD-1; even more, blocking exosome secretion alone or in combination with checkpoint blockade restores T cell effector function and increases survival and proliferation (Table 2) [12, 16, 17, 28].

Even when several studies have focused on how exosomal PD-L1 regulates CD8+ T cell effector function, further research is needed to address how exosomal PD-L1 may influence the development, differentiation, and function of other T cell subsets, such as regulatory T cells and the memory T cell pool. Evidence shows how exosomal PD-L1 regulates T cell function after acute activation *in vitro*; however, future studies could address how these observations relate to the different stages of T cell exhaustion both *in vitro* and *in vivo*.

PD-1 expression is not the unique feature of exhausted T cells; other inhibitory receptors such as LAG3, CTLA-4, and TIM-3 contribute to T cell exhaustion [48]. Further research is needed to thoroughly assess the contribution of additional inhibitory ligands in extracellular vesicles and how they correlate to PD-L1 function. Finally, T cell responses are a dynamic process that requires observation at different time points during tumor immune escape and tumor progression.

### 2.4. Exosomal Transfer of PD-L1 in the TME

Regardless of its physiological role in preventing autoimmunity, the expression of PD-L1 has been observed in tumor cells and tumor stroma, in particular cancer-associated fibroblasts (CAFs), tumor-associated macrophages (TAMs), myeloid-derived suppressor cells (MDSCs), and vascular endothelial cells (ECs) [65–67]. Expression of PD-1 is dependent on the context of the inflammatory microenvironment and can be observed in CD4+ and CD8+ T cells, B cells, macrophages, and dendritic cells [68–70].

As previously mentioned, there is evidence on the presence of PD-L1 mRNA and DNA enclosed in exosomes, as well as small RNA species that regulate the PD-L1 expression in recipient cells [13, 15, 71]. Functional PD-L1 can also be directly transferred from tumor cells to the cell surface of recipient cells upon exosome uptake in a dose-dependent manner [16]. In this context, tumor cells employ homotypical transfer of exosomes to modulate oncogenic signaling and promote tumor survival and progression [72, 73]. Likewise, the maintenance of the TME also appears to be mediated by the heterotypical transfer of exosomes which promotes paracrine signaling across multiple cell types [72].

**Figure 3:**
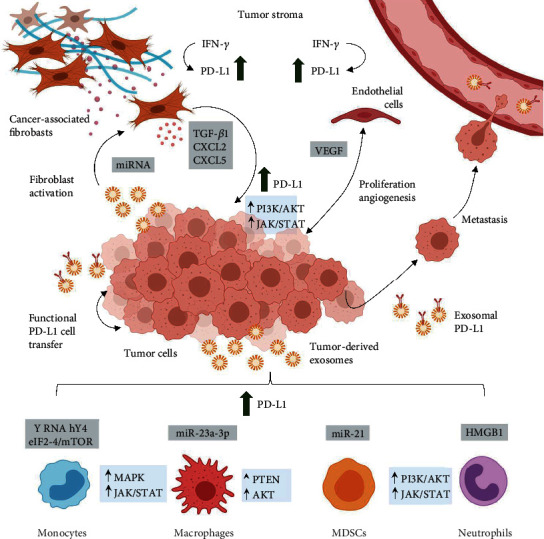
A macroscopic overview at the molecular crosstalk mediated by tumor-derived exosomes and exosomal PD-L1 in the TME. Exosomal PD-L1 mediates functional PD-L1 transfer between cells and induces systemic immunosuppression to facilitate metastasis. Tumor-derived exosomes induce the PD-L1 expression in monocytes, macrophages, myeloid-derived suppressor cells (MDSCs), and neutrophils via regulatory proteins and microRNA (gray boxes) which upregulate MAPK and JAK/STAT signaling (blue boxes). Cancer-associated fibroblasts and endothelial cells also express PD-L1 in response to interferon-*γ* (IFN-*γ*). Tumor-derived exosomes induce fibroblast activation which in turn results in the release of soluble factors that increase tumor PD-L1 expression. Likewise, tumor cells and endothelial cells interact to sustain a protumoral TME via soluble factors, such as vascular endothelial growth factor (VEGF).

The experimental observations show that tumors that are not responsive to immune checkpoint blockade exhibit increased exosomal PD-L1 accumulation in the TME and that blocking exosome release reverses therapy resistance and inhibits tumor growth [16]. However, the complex network of PD-L1 expression, function, and transfer mediated by exosomes is not completely understood.  Figure 3 shows a comprehensive overview of exosome-mediated PD-L1 transfer and upregulation across the TME. Considering these findings, further understanding of exosomal PD-L1 transfer in the TME is paramount to enhance immunotherapy response.

### 2.5. The Myeloid Compartment

Current studies continue to unveil how tumor cells induce phenotypic changes across immunocytes to establish and maintain a protumoral microenvironment [74]. Because of their outstanding plasticity to undergo functional polarization, monocytes and macrophages are primary targets of malignant transformation [75]. Tumor-associated macrophages (TAMs) have been associated with tumor progression through immunosuppression and the secretion of growth factors and thus are key contributors to the TME [76].

Haderk et al. demonstrated that tumor-derived exosomes induced an immunosuppressive phenotype in monocytes, which is characterized by the PD-L1 expression [71]. In this setting, chronic lymphocytic leukemia- (CLL-) derived exosomes transfer noncoding Y RNA hY4 to monocytes, inducing PD-L1 expression via toll-like receptor 7 (TLR-7) signaling [71]. Likewise, Liu et al. found that endoplasmic reticulum stress promotes hepatocellular carcinoma (HCC) cells to release exosomes that contained miR-23a-3p which upregulated PD-L1 expression in macrophages via the PTEN/AKT pathway [77].

Glioblastoma is another type of tumor that releases exosomal PD-L1 targeting monocytes which in turn suppress T cell function [78]. Additionally, glioblastoma-derived exosomes transfer protein components of the IFN-*γ*-JAK1/JAK2-STAT1/STAT2/STAT3-IRF1 signaling pathway to monocytes [79]. Gabrusiewicz et al. demonstrated that glioblastoma stem cell-derived exosomes exhibit an overrepresentation of proteins related to EIF2, eIF4/mTOR, ephrin receptor, and IGF-1 signaling which increase the phosphorylation of STAT3 and ERK1/2 in monocytes, inducing PD-L1 expression and ultimately inhibiting T cell tumor infiltration [79].

Extrinsic factors that induce PD-L1 expression in an exosome-mediated manner include hypoxia which drives tumor-derived exosomes to prime MDSCs to modulate *γδ* T cell activity via the miRNA-21/phosphatase and tensin homolog (PTEN)/PD-L1 axis [61]. In this setting, tumor-derived exosomes transfer small RNA species and critical regulatory proteins to induce a protumorigenic phenotype in myeloid-derived immunocytes, seizing the innate immune response and upregulating PD-L1 expression via canonical signaling.

Monocytes and macrophages are not the only immune cell subtypes prone to tumor-associated transformation [74]. Under physiological conditions, neutrophils can recruit macrophages which in turn modulate neutrophil function [76]. Evidence suggests that gastric cancer cell-derived exosomes can induce PD-L1 expression in neutrophils as well [80]. Shi et al. found that tumor-derived exosomes transported high-mobility group box-1 (HMGB1) to activate STAT3 and upregulate PD-L1 gene expression in neutrophils which in turn suppress T cell proliferation and function *in vitro* [80].

These findings strongly suggest that TAMs and tumor-associated neutrophils (TANs) cooperatively express PD-L1 to suppress T cell function and promote tumor progression. If TAMs and TANs release exosomal PD-L1 is unknown. Furthermore, the contribution of tumor-associated immune cells to the PD-L1 and exosomal PD-L1 burden in the TME is not known.

### 2.6. Tumor Stroma: Cancer-Associated Fibroblasts and Vascular Endothelial Cells

Immune infiltration analysis in solid tumors has revealed that PD-1 and PD-L1 expression is also associated with fibroblasts [81]. Cancer-associated fibroblasts (CAFs) comprise the majority of the tumor tissue in solid tumors and are critical modulators of the immune system in the TME [82]. Evidence suggests that CAFs express PD-L1 and also promote PD-L1 expression in tumor cells [83]. In this context, the relationship between stromal phenotype, PD-L1 status, and clinicopathological features has been proposed as a prognostic biomarker for patients with different molecular subtypes of breast cancer [83].

Current studies demonstrate that CAFs from a variety of tumor types release an array of factors that increase PD-L1 expression in tumor cells [84, 85]. Evidence suggests that TGF-*β*1, which is also found in CAF-derived exosomes, upregulates PD-L1 expression in a Smad2-dependent manner in NSCLC cells [86, 87]. M7824, a clinical-stage bifunctional compound that targets both PD-L1 and TGF-*β*, has been shown to limit malignant transformation and chemotherapy resistance in NSCLC [86]. It should be noted that developing novel compounds that target CAF-derived protumoral factors must also consider exosomal transfer in the TME.

CAFs secrete CXCL2 and CXCL5 which induce PD-L1 expression in tumor cells via JAK/STAT and PI3K/AKT signaling, respectively [82, 85]. These findings suggest that CAF-derived soluble factors upregulate PD-L1 expression in tumor cells by activating signaling pathways that have also been associated with exosomal PD-L1 release [84, 88, 89].

Evidence shows that PD-L1 mRNA and protein expression on CAFs derived from NSCLC is upregulated by exogenous supplementation with interferon-gamma (IFN-*γ*) which has shown to increase exosomal PD-L1 release in tumor cells [90]. Treatment of CAFs with GW4869, an exosome release inhibitor, significantly reduces chemotherapeutic drug resistance in pancreatic ductal adenocarcinoma cells (PDAC) [91]. These findings suggest a strong pragmatic rationale to inhibit CAF exosome release to enhance immunotherapy efficacy. However, the CAF release of functional exosomal PD-L1 is not known.

Finally, recent evidence suggests that vascular endothelial cells (EC) also express PD-L1 after IFN-*γ* stimulation and thus are resistant to apoptosis mediated by T cells [92, 93]. However, the EC release of exosomal PD-L1 is unknown. Intercellular communication between EC and malignant cells induces proliferation in both cell types, sustaining angiogenesis and promoting metastasis [94]. EC release exosomes to mediate cardiovascular tissue regeneration; nonetheless, the role of EC-derived exosomes in the TME is not completely understood [95]. For instance, the immunomodulatory properties of EC-derived exosomes have not been thoroughly described.

On its part, vascular endothelial growth factor (VEGF) is a critical factor for endothelial cell transformation and has been related to the expression of inhibitory receptors on T cells [96]. Thus, there appears to be an association between immunomodulation and proangiogenic transformation in the setting of tumor development and progression. Even more, data suggests that inhibiting both VEGF and PD-L1 can be an effective combinatorial strategy in small cell lung cancer (SCLC) [96]. Pivotal questions to be addressed concern the influence of EC-derived exosomes in immune escape and immunotherapy.

Interestingly, PD-L1 signaling in tumor cells has also been explored. Signaling motifs associated with the cytoplasmatic domain of PD-L1 have not been found. However, evidence suggests that PD-L1 binding to PD-1 may induce tumor cell resistance to chemotherapy, enhanced glycolytic metabolism, and greater migration and invasion capacities in the absence of T cells [2, 31, 97]. If compelling evidence arises, the release of exosomal PD-L1 by tumor and tumor-associated cells could be considered as a mechanism that sustains the TME and facilitates premetastatic niche formation, beyond immune escape.

## 3. Exosomal PD-L1 as a Biomarker for Cancer Immunotherapy

### 3.1. Diagnosis, Prognosis, and Staging

Given their outstanding clinical efficacy, immune checkpoint inhibitors have become the first-line treatment for various advanced tumors, yet therapy response rates are low [98]. Individual variability and tumor heterogeneity are factors proposed to hinder immunotherapy response, and identifying those patients most likely to benefit from therapy is crucial for a personalized approach [81]. Indeed, progress in molecular pathology has revealed that adequate pathological methods and molecular testing significantly improve molecular-directed cancer therapies [99]. Even more, an accurate diagnosis, staging, and prognosis are ultimately required to improve immunotherapy response.

In this context, the identification of tumor PD-L1 expression by immunohistochemistry (IHC) is the most widely used biomarker for predicting immunotherapy response [65, 81, 100]. Nonetheless, methodological variations among studies, as well as the dynamic regulation of PD-L1 expression, have resulted in confounding evidence, which limits the detection of tumor PD-L1 expression as an exclusionary biomarker [100]. On this basis, the concept of liquid biopsy may reemerge as a noninvasive tool for screening candidate factors that may influence the clinical outcome of immunotherapy.

The first attempts to study extracellular PD-L1 as an immune biomarker focused on soluble PD-L1 at large, which means that there was no initial distinction between circulating PD-L1 in exosomes, microvesicles, or its membrane-free soluble forms: monomeric, dimeric, and splice variants [29, 30]. In these early studies, the contribution of exosomal PD-L1 to immunosuppression or immunotherapy response was not specifically assessed. Yet, identifying circulating PD-L1 components is not trivial, since the evidence shows that dimeric soluble PD-L1 elicits an immunosuppressive function both *in vitro* and *in vivo* [101].

In this framework, circulating soluble PD-L1 has proved to be elevated in advanced NSCLC patients when compared to healthy controls [102]. In the same study, the level of circulating soluble PD-L1 was used to divide NSCLC patients into high and low expression groups; higher levels were positively correlated with metastasis and a worse prognosis [102]. Likewise, Zhou et al. showed that pretreatment levels of soluble PD-L1 were also elevated in metastatic melanoma patients when compared to healthy donors; however, it could only be used as a biomarker of progressive disease in a subgroup of patients [29].

In contrast, higher soluble PD-L1 levels correlated to a much better prognosis and the absence of lymph node metastasis in gastric adenocarcinoma (GC) [103]. Nonetheless, soluble PD-L1 levels could not be correlated to the degree of tumor differentiation or any other clinicopathological variable in the same study [103]. Likewise, Fan et al. found no correlation between the levels of soluble PD-L1 prior to surgery and clinical outcome in GC [58]. The aforementioned studies suggest that higher levels of circulating PD-L1 indicate the presence of malignancy. However, the level of soluble PD-L1 could not be consistently correlated to tumor staging and prognosis, resembling the detection pattern of tumor PD-L1 expression by IHC.

Considering the role of exosomes in cancer biology, the current research is aimed at determining the value of exosomal PD-L1 to predict immunotherapy response and clinical outcome (Table 3). Certainly, the circulating level of exosomal PD-L1 may reflect both, an immunosuppressive TME and the immune status of the patient [12, 58, 59]. Even more, considering that PD-L1 in exosomes has also been related to tumor growth and progression, exosomal PD-L1 is not only a prime candidate to be used as a predictive biomarker of therapy response, but it may aid in the diagnosis and staging of cancer [14, 62].

Exosomal PD-L1 provides a platform to assess the immune status of cancer patients employing noninvasive methods that can be performed at different points in time. However, correlating the level of exosomal PD-L1 to tumor PD-L1 expression by IHC remains a challenge. For instance, exosomal PD-L1 in plasma of patients with NSCLC was shown to strongly correlate to tumor PD-L1 positivity by IHC [28]. Conversely, recent studies showed that exosomal PD-L1 does not correlate to tumor PD-L1 detection in melanoma and NSCLC patients [62, 104]. Likewise, further research into the dynamic regulation of PD-L1 expression is required.

Regardless of an inconsistent association between tumor PD-L1 expression and the level of circulating exosomal PD-L1, evidence shows that exosomal PD-L1 is higher in patients with metastatic melanoma than in healthy donors [12]. Even more, the level of exosomal PD-L1 positively correlated with overall tumor burden and IFN-*γ* levels, indicating a poor prognosis [12]. The same study showed that the levels of other forms of soluble PD-L1 could not distinguish melanoma patients from healthy donors [12].

In the same way, exosomal PD-L1 was shown to distinguish NSCLC patients from healthy donors [104]. Furthermore, the level of exosomal PD-L1 correlated to tumor size, lymph node status, and metastasis, while soluble PD-L1 did not correlate to any clinicopathological feature in NSCLC patients [104]. Likewise, in head and neck squamous cell carcinoma (HNSCC), the level of exosomal PD-L1 correlated to disease activity [64]. Patients with advanced disease showed higher levels of exosomal PD-L1 than patients that had no evidence of disease after completing curative therapy, or even than patients in stage I and II [64]. In the same study, soluble PD-L1 did not correlate to any clinicopathological feature [64].

Lux et al. showed that in patients with PDAC, higher levels of exosomal PD-L1 correlated to an unresectable tumor at the time of diagnosis and to a decrease in the median postoperative survival time [105]. Likewise, exosomal PD-L1 was found to be an independent prognostic factor for early-stage gastric adenocarcinoma, while it was also associated with tumor stage [58]. In this study, higher levels of exosomal PD-L1 before surgery was associated with a worse clinical outcome and reflected immunosuppression as demonstrated by a lower CD4+ and CD8+ T cell count, decreased granzyme B, and increased IL-10 and TGF-*β* levels before chemotherapy [58].

Thus far, evidence suggests that exosomal PD-L1 may be a superior cancer biomarker for diagnosis, staging, and prognosis than other forms of soluble PD-L1. It should be noted that EVs transport soluble proteins and genetic material along with surface molecules, which highlights the value of exosomes as a potential indicator of disease activity. For instance, it was recently found that although exosomal PD-L1 levels could not distinguish glioblastoma patients from healthy donors, exosomal PD-L1 DNA enrichment correlated to tumor volume of up to 60 cm^3^, suggesting that PD-L1 DNA in exosomes reflects tumor burden [15].

Given these findings, we can summarize that higher levels of exosomal PD-L1 have been associated with advanced disease and a worse prognosis in melanoma, NSCLC, HNSCC, PDAC, gastric cancer, and glioblastoma. Also, higher levels of exosomal PD-L1 have been found in patients with advanced disease when compared to healthy controls. In this context, exosomal PD-L1 appears to be a more consistent biomarker than soluble PD-L1. These findings provide a rationale to further develop exosomal PD-L1 as a diagnostic and prognostic cancer biomarker.

In this setting, the level of circulating PD-L1 may thus reflect the immune status of the patient and disease activity at the time of assessment. Higher levels of exosomal PD-L1 correlate to a highly immunosuppressive tumor able to reach distant tissues, which is by itself an indicator of late-stage and poor prognosis [2, 10]. Even more, since the PD-L1/PD-1 signaling axis has also been associated with tumor growth and premetastatic niche formation, the level of exosomal PD-L1 may reflect the proliferative capacity and invasiveness of tumor cells [20, 73].

Nonetheless, PD-L1 expression is dynamic and influenced by many factors, and thus, measuring other immune biomarkers along with exosomal PD-L1 can be useful to better correlate the level of circulating PD-L1 with the immune status of the patient [51, 59]. Even more, other immune checkpoints can also mediate tumor immune escape [5, 8, 106]. Exploring the relative contribution of other immunomodulatory molecules to tumor immune escape is also relevant to advance the use of exosomal PD-L1 as a biomarker.

It should be noted that tumor cells may not be the only source of exosomal PD-L1 and that aging and other chronic inflammatory conditions have also been associated with PD-L1 expression [107–109]. Therefore, exploring the physiology of exosomal PD-L1 in other inflammatory conditions and across cell types is important since it may ultimately result in the full development of exosomal PD-L1 as a cancer biomarker.

Future studies must focus on establishing cutoff values to distinguish subgroups of patients and standardizing detection techniques. In this context, correlating the level of exosomal PD-L1 to established factors associated with cancer progression such as tumor mutational burden and differentiation can be paramount to clarify discrepancies among studies [99, 107].

### 3.2. Prediction of Immunotherapy Response and Therapeutic Efficacy

In clinical practice, the use of circulating biomarkers has the potential to predict the clinical outcome and to assess treatment response, which is urgent to expand the benefits of immune checkpoint blockade in cancer. Exosome research is an emerging field, and thus, evidence is limited and often contrasting. Certainly, to thoroughly assess the clinical implications of exosomal PD-L1 in cancer immunotherapy, further research is required to integrate the knowledge on exosomal PD-L1 biogenesis and physiology to clinical observations.

Zhou et al. showed that a higher concentration of soluble PD-L1 before immunotherapy correlated to progressive disease in a subgroup of patients with metastatic melanoma [29]. In the same setting, measuring the concentration of soluble PD-L1 early after PD-1 or CTLA-4 blockade was not useful to distinguish responders from nonresponders [29]. Likewise, baseline levels of exosomal PD-L1 are not consistently associated with clinicopathological features of advanced cancer patients (Table 3).

However, changes in exosomal PD-L1 after immunotherapy correlate to overall survival (OS) and progression-free survival (PFS) in melanoma patients treated with immune checkpoint blockade [62]. Cordonnier et al. recently showed that a decrease in exosomal PD-L1 levels after PD-1 blockade is associated with complete or partial responses, while an increase in such levels results in disease progression [62]. Furthermore, evidence also shows that a higher baseline level of circulating exosomal PD-L1 in metastatic melanoma patients correlates to a worse clinical outcome after immunotherapy with pembrolizumab [12]. Clinical responders showed increased levels of exosomal PD-L1 after 6 weeks of therapy, which was preceded by an increase in CD8+ T cell proliferation [12]. These findings indicate that a fold change in the level of exosomal PD-L1 greater than 2.43 after pembrolizumab correlates to a better clinical outcome, highlighting the importance of systematically defining the cutoff values or thresholds [12].

Zhang et al. recently showed that the expression of soluble PD-L1 in the plasma of patients with a variety of cancer types before PD-1 blockade therapy could not be used to predict therapy response [59]. However, higher exosomal PD-L1 levels were a negative prognostic factor after PD-1 blockade, while an increase in exosomal PD-L1 was associated with a better clinical outcome after PD-1 blockade in different types of cancers [59].

As noted by the aforementioned studies, there is no concordance among available data to assume that a higher or a lower exosomal PD-L1 level before immunotherapy results in complete or partial responses or in a better clinical outcome (Table 3). Arguably, evidence suggests that higher baseline exosomal PD-L1 levels correlate to a worse clinical outcome. Likewise, conflicting results have also been found when assessing the change in exosomal PD-L1 level after immunotherapy. However, most studies show that an increase in exosomal PD-L1 level indicates complete or partial responses and a better prognosis.

As we have previously noted, higher levels of exosomal PD-L1 have been associated with advanced disease and poor prognosis regardless of the intention to treat with checkpoint blockade [64, 104]. In this context, higher levels of exosomal PD-L1 may be associated with immunotherapy failure because the immune response may have already reached a level of immunosuppression that is beyond reinvigoration [12]. For instance, the comprised expression of tumor PD-L1 and exosomal PD-L1 release may challenge antibody blockade. Exosomal PD-L1 may mediate therapy failure by binding anti-PD-L1 antibodies, engaging them in both the systemic circulation and the TME [12]. Even more, since higher exosomal PD-L1 levels at baseline may reflect a highly immunosuppressive tumor, other immune checkpoints and immunosuppressive molecules may also sustain immunosuppression after PD-L1/PD-1 blockade [106].

An increase in exosomal PD-L1 levels after immunotherapy may hint adaptive tumor responses and thus correlate to complete or partial responses [12]. Conversely, evidence also shows that a decrease in exosomal PD-L1 levels is associated with a better clinical outcome after immunotherapy; from that perspective, a decrease in tumor burden may also cause a decrease in extracellular PD-L1 release [62]. These findings certainly highlight the urgent need to track the evolution of exosomal PD-L1 at different time points after therapy, since the discrepancies observed among studies may be dependent on the time of testing and thus may reflect the evolution of tumor immune escape.

Remarkably, recent evidence suggests that the number of copies per milliliter of PD-L1 mRNA in plasma-derived exosomes can be used to assess immunotherapy response and disease progression in melanoma and NSCLC [13]. Higher pretreatment levels of exosomal PD-L1 mRNA were associated with partial and complete responses to pembrolizumab and nivolumab, while a decrease in the number of copies per milliliter of exosomal PD-L1 after treatment was also associated with complete and partial responses [13]. Conversely, an increase in exosomal PD-L1 mRNA after therapy was associated with progressive disease [13].

Overall, because of the lack of compelling and consistent evidence, the use of exosomal PD-L1 as a cancer biomarker is still not suitable for clinical practice. However, measuring exosomal PD-L1 to define which cancer patients may benefit from checkpoint blockade, as well as after treatment, to assess therapeutic efficacy is an attractive complementary diagnostic assay that can aid in the risk-benefit analysis. Even more, if further research is conducted, it may ultimately lead to companion diagnostic assays to validate exosomal PD-L1 as a predictive biomarker to direct treatment decisions.

## 4. Concluding Remarks and Future Directions

The study of immune checkpoints in cancer has led to the development of novel therapeutic opportunities, such as PD-L1/PD-1 blockade. Immune checkpoint inhibitors have demonstrated an outstanding clinical efficacy in a variety of advanced tumors which reveals that amidst a complex and dynamic TME, cancer is vulnerable to the immune response. Nonetheless, the TME is in constant evolution and thus restrains immunotherapy efficacy.

Clinical observations have associated extracellular forms of PD-L1 to low response rates and therapy resistance. In particular, exosomal PD-L1 has emerged as a pivotal mechanism of immune escape and tumor progression. The recognition of exosomal PD-L1, which can be targeted experimentally and clinically, has been a paramount breakthrough with the prospective to revolutionize immunotherapy for cancer and chronic inflammatory conditions.

Regardless of this prospective, we know little about the molecular mechanisms that result in exosomal PD-L1 release. Even more, we require a further understanding of the immunoregulatory function of exosomal PD-L1 and of when and how, in the context of tumor development and progression, exosomal PD-L1 initiates and elicits its immunoregulatory role. Therefore, a paramount goal is to answer these unsolved questions to improve immunotherapy response and efficacy.

Moreover, we still need a further molecular understanding of how exosomal PD-L1 is transferred across cancer and cancer-associated cells and the influence of this form of extracellular PD-L1 on systemic immunity. Although both tumor-derived exosomes and immune cell-derived exosomes are critical modulators of the immune response, intensive research has been dedicated to the physiology of tumor-derived exosomes, and thus, the contribution of immunocytes to the exosomal burden in the TME remains to be elucidated.

A major defining feature of exosomal PD-L1 is its role in tumor growth and metastasis; therefore, elucidating both the intracellular signalosome of PD-L1 and PD-1 across tissues is paramount. Certainly, studying several other critical aspects of the PD-L1/PD-1 axis will reveal new insights into fundamental aspects of immune escape and tumor progression, including novel therapeutic targets. In this context, continuous study of exosomal PD-L1 biogenesis, release, and function is crucial.

It appears that targeting nontumor cells or mediators of their communication is a feasible strategy to limit cancer progression. Tumor-derived exosomes are recognized as a powerful driver for tumor progression, and thus, they are a formidable target for treatment and a promising prognostic biomarker, yet translating the knowledge of experimental and clinical observations to effective treatment still requires further work.

In this work, we have discussed the latest evidence on exosomal PD-L1 biogenesis, function, and transference across the TME, including the factors that drive its synthesis and the mechanisms by which it elicits its immunomodulatory effects. We have also reviewed the dynamic subcellular distribution of PD-L1 in the context of exosome release, and the relationship between exosomal PD-L1 and critical aspects of cancer immunotherapy.

According to the latest studies, exosomal PD-L1 arises from the plasma membrane and remains with the same topography as it is loaded into exosomes for extracellular release. PD-L1 has been colocalized with proteins associated with intravesicular transport, early and late endosome biogenesis, and exosomal release. Although exosomal PD-L1 has been related to PD-L1 expression, the precise factors that drive exosomal PD-L1 release remain to be known. Remarkably, there appears to be a dynamic relationship between the cell surface and exosomal PD-L1, and elucidating the factors and mechanisms that influence PD-L1 subcellular localization could improve the use of exosomal PD-L1 as a biomarker and target for cancer immunotherapy.

Upon release, exosomal PD-L1 effectively binds to the PD-1 receptor. Current evidence suggests that exosomal PD-L1 elicits the same function as its cellular counterpart, terminating T cell activation and sustaining an exhausted phenotype which ultimately results in an immunosuppressive environment, both locally and systemically. Exosomal PD-L1 appears to inhibit T cell effector function. Nevertheless, the effect of exosomal PD-L1 on the array of cells that express PD-1 and the context of these interactions remains to be further explored.

As mentioned, PD-L1 has been strongly related to malignant transformation. Here, we have reviewed the latest studies that indicate that exosomes are a suitable vehicle to transfer functional PD-L1 or critical regulatory proteins and genetic material that upregulate PD-L1 expression in recipient cells. However, much remains to be done to understand the factors that drive exosome-mediated PD-L1 transfer in the TME and its role in tumor progression.

The intricate network of PD-L1 transfer across the TME adds to the theory of a dynamic microenvironment in which adaptive tumor responses drive malignant transformations to ensure survival and growth. Studying the role of the exosomal PD-L1 in the physiology of TAMs, TANs, CAFs, and ECs is of great interest to improve cancer treatment. Furthermore, measuring the level of exosomal PD-L1 can be an effective complementary diagnostic assay to define which patients may benefit from checkpoint blockade and to assess therapeutic efficacy.

In short, exosomal PD-L1 appears to be a critical mechanism for immune escape and metastasis, and it may have major implications in immunotherapy. Tracking the evolution of exosomal PD-L1 in tumor establishment and progression may contribute to the diagnosis of cancer and the prediction and assessment of therapeutic interventions. Lastly, novel therapeutic strategies are being developed to restrain exosome secretion and function, promising a new paradigm shift in cancer treatment.

## Figures and Tables

**Table 1:** PD-L1 subcellular distribution and associated regulatory factors.

Cancer type	Regulatory factor	Exosomal PD-L1	Cell surface PD-L1	Reference
Melanoma	IFN-*ɣ*	**↑**	**↑**	[12]
Melanoma	HRS knockdown	**↓**	**↑**	[12]
Melanoma	IFN-*α*, IFN-*ɣ*, TNF-*α*	**↑**	**↑**	[29]
Glioblastoma	IFN-*ɣ*	**↑**	**↑**	[15]
BC	ALIX knockdown	**↓**	**↑**	[25]
NSCLC	Acquired TKI resistance	**↑**	**↑**	[28]
PC	Rab27a knockdown	**↓**	**=**	[17]
PC	nSMase2 knockdown	**↓**	**↓**	[17]

Subcellular PD-L1 expression includes: **↑** promotion, ↓ inhibition, = unchanged. IFN-*γ*: interferon-*γ*; IFN-*α*: interferon-*α*; HRS: hepatocyte growth factor-regulated tyrosine kinase substrate; TNF-*α*: tumor necrosis factor-*α*; BC: breast cancer; ALIX: ALG-2 interacting protein X; NSCLC: non-small-cell lung cancer; TKI: tyrosine kinase inhibitors; PC: prostate cancer; Rab27a: Ras-related protein 27a; nSMAase2: neutral sphingomyelinase 2.

**Table 2:** Immunological effects of blocking exosomal PD-L1.

Cancer type	Experimental setting	Effect	Reference
HNSCC	Exosomal PD-L1 was isolated from the plasma of HNSCC patients and coincubated with activated CD8 + T cells ± PD − 1 inhibitor.	Exosomal PD-L1 downregulates CD69 expression which is upregulated after PD-1 blockade.	[64]
GC	Exosomal PD-L1 was isolated from the CCM of MNK74 cells and coincubated with Jurkat T cells and PBMC ± Nivolumab.	Exosomal PD-L1 induces Jurkat T cell apoptosis and downregulates CD69 and CD25 expression in PBMC; both effects were reversed after PD-1 blockade.	[41]
Glioblastoma	Exosomal PD-L1 was isolated from CCM of PCC derived from glioblastoma patients and coincubated with activated CD4+ and CD8 + T cells ± PD − 1 antibodies.	PD-1 blockade restores T cell activation as measured by CD69 and CD25 expression.	[15]
Melanoma	Exosomal PD-L1 isolated from PD-L1/MEL624 cells was preincubated with anti-PD-L1 antibodies and then incubated with activated CD8+ T cells.	Exosomal PD-L1 blockade restored T cell proliferation, granzyme B, IFN-*γ*, IL-2, and TNF-*α* production.	[12]
NSCLC	Exosomal PD-L1 isolated from H460 and H1975 cells was preincubated with anti-PD-L1 antibodies and then incubated with activated Jurkat T cells.	Exosomal PD-L1 decreased the IFN-*γ* production in a dose-dependent manner, while PD-L1 blockade restored IFN-*γ* secretion.	[28]

HNSCC: head and neck squamous cell cancer; GC: gastric cancer; CCM: cell culture medium; PBMC: peripheral blood mononuclear cells; PCC: primary cell culture; IFN-*γ*: interferon-*γ*; TNF-*α*: tumor necrosis factor-*α*; IL-2: interleukin-2; NSCLC: non-small-cell lung cancer.

**Table 3:** Clinical implications of exosomal PD-L1 in cancer diagnosis, prognosis, and immunotherapy response.

Tumor type (*n*)	Aim	Outcome	Reference
Soluble PD-L1			
Melanoma (35)	To compare the serum concentration of soluble PD-L1 in melanoma patients and healthy donors and to explore the clinical significance of soluble PD-L1 in patients with melanoma on PD-1 blockade.	The level of soluble PD-L1 was elevated in metastatic melanoma patients when compared to healthy donors (*P* = 0.004). Higher baseline levels (≥1.4 ng/mL) of PD-L1 were associated with PD in patients treated with PD-1 blockade (*P* = 0.001). After 5 months of treatment with PD-1 blockade, a ≥1.5-fold increase in circulating PD-L1 level correlated to a PR (*P* = 0.007).	[29]
NSCLC (109)	To compare the mean level of circulating PD-L1 in NSCLC patients and healthy controls and to evaluate the association between the level of serum-derived soluble PD-L1 and the clinical characteristics of advanced NSCLC patients.	The mean level of PD-L1 in NSCLC patients (0.723 ± 0.081 ng/mL) was significantly higher when compared to healthy controls (0.565 ± 0.048 ng/mL) (*P* < 0.001). A cutoff value of 0.636 ng/mL distinguished patients in the high and low expression groups. Higher PD-L1 expression correlated to abdominal organ metastasis (*P* = 0.004). The median OS in patients of the low expression group (26.8 months) was longer than in the high group (18.7 months) (*P* < 0.001).	[102]
GC (80)	To compare the expression of circulating PD-L1 in advanced GC patients to healthy controls and to evaluate the association between serum-derived PD-L1 and the prognosis of patients with advanced gastric cancer.	The mean value of circulating PD-L1 level in GC patients (0.8928 ± 0.0900 ng/mL) was higher than in healthy controls (0.5899 ± 0.0617 ng/mL) (*P* = 0.006). A cutoff value of 0.5993 ng/mL distinguished GC patients in high and low upregulated PD-L1 groups (*P* = 0.04). The OS 5-year rate in the high PD-L1 group was 65.6% and 44.7% in the low group (*P* = 0.028). High soluble PD-L1 expression was associated with GC differentiation (*P* = 0.032) and the absence of lymph node metastasis (*P* = 0.041).	[103]
Exosomal PD-L1			
Melanoma (44)	To compare the level of plasma-derived exosomal PD-L1 in melanoma patients to healthy donors and to correlate the level of exosomal PD-L1 with the clinical response to pembrolizumab.	The level of exosomal PD-L1 was ∼5 times higher in patients with metastatic melanoma than in healthy donors (*P* = 0.0002). A cutoff value of 1.03 ng/mL of exosomal PD-L1 distinguished responders (low) from nonresponders (high) to pembrolizumab therapy (*P* = 0.0018). A fold change > 2.43 ng/mL in exosomal PD-L1 levels at weeks 3-6 after pembrolizumab correlated to prolonged PFS and OS up to 15 months after landmark (*P* = 0.00002), with a sensitivity of 80% and specificity of 89.47%.	[12]
HNSCC (40)	To evaluate the potential contributions of plasma-derived exosomal PD-L1 to disease activity in patients with HNSCC.	Higher percentages of exosomal PD-L1 were observed in patients with AD compared to NED (*P* < 0.0137). Patients with N1 disease had significantly higher percentages of exosomal PD-L1 than those who were N0 (*P* < 0.0008). Patients in stages III and IV had higher percentages of exosomal PD-L1 than patients in stage I/II (*P* < 0.0001). The average level of soluble PD-L1 in plasma was 53.6 pg/mL ± 50.8, which did not correlate to any clinicopathological data.	[64]
NSCLC (24)	To correlate the level of plasma-derived circulating exosomal PD-L1 to PD-L1 expression in tumor tissue of patients with NSCLC prior to surgical resection.	The number of PD-L1 positive exosomes strongly correlated with the level of PD-L1 expression in tumor tissue as measured by IHC (*P* = 0.0367). However, the proportion of PD-L1 positive exosomes from each patient varied between 10 and 70%.	[28]
PDAC (17)	To assess whether pancreatic carcinoma releases exosomal PD-L1 when compared to SCA and CP and whether the detection of such expression has diagnostic or prognostic value in PDAC patients.	The level of exosomal PD-L1 in patients' serum was not able to distinguish PDAC patients from CP patients. A cutoff value of >299 was used to distinguish positive or negative exosomal PD-L1 levels. Exosomal PD-L1 positivity was correlated to an unresectable tumor at the time of diagnosis (*P* = 0.01). The median postoperative survival of exosomal PD-L1-negative patients was significantly longer (17.2 months) than exosomal PD-L1-positive patients (7.8 months) (*P* = 0.043).	[105]
NSCLC (85)	To investigate the clinical significance of serum-derived exosomal PD-L1 in NSCLC and to explore the correlation between exosomal PD-L1 expression and PD-L1 expression in tumor tissue of NSCLC patients.	Levels of exosomal PD-L1 levels of stage I-II (15.90 ± 6.45 pg/mL) and III/IV NSCLC patients (21.10 ± 11.63 pg/mL) were considerably higher than that of healthy controls (15.91 ± 6.45 pg/mL) (*P* < 0.05 and *P* < 0.001, respectively). Higher levels of exosomal PD-L1 were associated with advanced tumor stage (II, III, IV, *P* = 0.012), tumor size > 2.5cm (*P* = 0.003), lymph node status N1-3 (*P* = 0.03), and M1 (*P* = 0.026). Exosomal-PD-L1 levels did not correlate to PD-L1 IHC profiles.	[104]
Melanoma (100)	To evaluate the use of plasma-derived exosomal PD-L1 of melanoma patients to predict immunotherapy response and clinical outcome and to study the association between exosomal PD-L1 expression, tumor PD-L1 IHC detection, and soluble PD-L1.	The mean level of exosomal PD-L1 (64.26 pg/mL) was higher when compared with soluble PD-L1 (30, 0.1 pg/mL) and tumor positivity (100 vs. 67%) at baseline. The baseline level of exosomal PD-L1 was not associated with any clinicopathological feature. In patients with PD after immunotherapy, exosomal PD-L1 increased significantly (85.90 ± 24.4 vs. 344.20 ± 70.30, *P* = 0.0002). Patients experiencing CR and PR showed a decrease in exosomal PD-L1 after PD-1 blockade (*P* = 0.001). A cutoff value < 100 showed that a decrease in exosomal PD-L1 was associated with a better clinical outcome as measured by PFS and OS (*P* = 0.048 and 0.001, respectively).	[62]
GC (69)	To evaluate the prognostic value of plasma-derived exosomal PD-L1 in gastric cancer patients before surgery.	A cutoff value of 82.585 ng/mL of exosomal PD-L1 was used to analyze the correlations and survival analysis. OS was significantly lower in the high exosomal PD-L1 group than in the low group in patients with stage I and II (*P* = 0.004). Higher exosomal PD-L1 was associated with an advanced T stage (*P* = 0.028) and lymphatic invasion (*P* = 0.014).	[58]
NSCLC (25), SCLC (2), GC (1), HNSCC (3), CC (2), RCC (1), HCC (1), CHC (2), EC (5), DC (1) and melanoma (1)	To investigate the role of plasma-derived exosomal PD-L1 as a predictive biomarker and to assess therapy efficacy with PD-1 blockade in a variety of cancer types.	Exosomal PD-L1 of the NR was significantly higher than the R before treatment (*P* = 0.010). In the NSCLC cohort, low levels of exosomal PD-L1 before PD-1 therapy correlated to prolonged PFS (2 vs. 8 months, *P* = 0.010). Exosomal PD-L1 and tumor burden decreased when the therapy was effective (*P* < 0.005).	[59]
Genomic exosomal PD-L1			
Melanoma (18), NSCLC (8)	To investigate the association between PD-L1 mRNA in plasma-derived exosomes and response to nivolumab and pembrolizumab treatment in patients with melanoma and NSCLC.	A higher number of exosomal PD-L1 mRNA (830.4 ± 231.3 copies per mL) before therapy was positively associated with CR and PR, compared to patients with SD (298.8 ± 97.2 copies per mL) or PD (204.0 ± 68.8 copies per mL). The number of mRNA copies per milliliters of exosomal PD-L1 mRNA decreased in patients with CR (242.5 ± 82.5, *P* = 0.016), while it increased in the case of PD (416.0 ± 87.8, *P* = 0.001).	[13]
Glioblastoma (21)	To correlate serum- and plasma-derived exosomal PD-L1 DNA to tumor burden in patients with glioblastoma.	Enrichment of circulating exosomal PD-L1 DNA distinguished healthy controls from glioblastoma patients, while it also correlated to tumor volume (*P* = 0.0025). There was also a positive correlation between the PD-L1 expression in glioblastoma tissue and circulating exosomal PD-L1 DNA (*P* = 0.01).	[15]

PD: progressive disease; CR: complete response; PR: partial response; NR: nonresponders; R: responders; SD: stable disease; AD: advanced disease; NED: not established disease; OS: overall survival; PFS: progression-free survival; IHC: immunohistochemistry; NSCLC: non-small-cell lung cancer; HNSCC: head and neck squamous cell carcinoma; PDAC: pancreatic ductal adenocarcinoma; CP: chronic pancreatitis; SCA: serous cystadenoma of the pancreas; SCLC: small cell lung cancer; CC: colon cancer; HCC: hepatocellular carcinoma; RCC: renal cell carcinoma; CHC: cholangiocarcinoma; DC: duodenal carcinoma; EC: esophageal carcinoma; (*n*): number of patients.
